# Prevalence of acute kidney injury in Mexico; a systematic review and meta-analysis of pre-pandemic reports

**DOI:** 10.1080/0886022X.2024.2449573

**Published:** 2025-01-30

**Authors:** Jose J. Zaragoza, Jonathan S. Chavez-Iñiguez, Armando Vazquez-Rangel

**Affiliations:** ^a^ Critical Care Department, Hospital H + Queretaro, Qro., Mexico, Queretaro, Mexico; ^b^ Nephrology Department, Hospital Civil de Guadalajara Fray Antonio Alcalde, Guadalajara, Jal, Mexico; ^c^ Nephrology Department, Instituto Nacional de Cardiologia, Mexico City, Mexico

**Keywords:** Acute kidney injury, mortality, kidney replacement therapy, epidemiology

## Abstract

**Background:**

Acute Kidney Injury (AKI) is a health problem worldwide, accounting for high hospital morbidity and mortality. There is little available information regarding the characteristics and incidence of AKI in Latin America (LA), especially in Mexico.

**Objectives:**

Systematically search the literature and perform a meta-analysis of the epidemiology of AKI in Mexico, to provide data on AKI and kidney replacement therapy (KRT) that would contribute to general knowledge in this matter.

**Methods:**

We performed a systematic search for articles in pediatric and adult patients admitted to the general ward, Emergency Room or Intensive Care Unit published between January 1, 2000, and September 30, 2024. MEDLINE LILIACS, EMBASE and SciELO were searched, as additional reports from supplements, abstracts, and conference sessions. We performed a random-effects meta-analysis for clinically and methodologically comparable studies to estimate the frequency of AKI and KRT. We calculated pooled estimates stratified by age group, year of publication, and setting.

**Results:**

83 full-text articles were included. The percentage of AKI was calculated at 35% (95% CI, 28–42). Mortality for AKI adult patients was 36% (95% CI, 28–45). An overall KRT rate was 7% (95% CI, 6–9), all-cause mortality for AKI requiring KRT was 49% (95% CI, 42–56), with a global Ι2 estimated in 99.87% (*p* < 0.01).

**Conclusion:**

AKI is common in Mexico and remains a main public health problem that needs to be addressed at every level of care. Efforts should be made to reinitiate AKI research and control in Mexico and LA.

## Introduction

Acute Kidney Injury (AKI) is a serious health problem worldwide, that accounts for high hospital morbidity, mortality, and care-related costs [1,2]. Recent epidemiological and observational studies have indicated the relevance of AKI, not only as an acute syndrome but also as a mediator and/or marker of long-term adverse outcomes [3].

It has been difficult to assess AKI epidemiology accurately since a definition has been only recently standardized, and data are still reported according to different classifications (RIFLE, AKIN and Kidney Disease Improving Global Outcomes [KDIGO]), with only a limited number of studies including urine output criteria into the inclusion criteria [4]. In addition, the general use of biomarkers is not yet spread for clinical care and is mainly used for research purposes [4]. Hence, is common to find high heterogeneity among published papers, with a high imbalance of information and practices between the northern and southern hemispheres and between high and low-middle-income countries [5]. Another historical problem in collecting information about the epidemiology of AKI in Latin America has been the lack of standardization of the term AKI when using synonyms such as insufficiency, damage, or failure.

In this context, the main goal of the 0 by 25 initiative from the International Society of Nephrology is to eliminate (or at least reduce) avoidable AKI-related deaths worldwide by 2025. The aim number one of this project is to establish the burden of AKI through epidemiological reports with standardized definitions and consider a key point on the research agenda for AKI epidemiological data accumulation [6].

It has been postulated that AKI has a bimodal pattern [3]. In urban areas, AKI characteristics are like those found in high-income countries: it is a predominantly hospital-acquired disease affecting mostly older, critically ill multiorgan failure patients with previous comorbidities, and it is caused mainly by sepsis or in association with nephrotoxic drugs [3]. In remote, rural and/or poorer areas, AKI affects mainly young (30–40 years) and previously healthy individuals, with a profile strongly influenced by environmental conditions, infectious diseases, exposure to animal venom, septic abortion, and exposure to herbs and compounds used in traditional medicine [3,7].

Unfortunately, there is little available information regarding the characteristics and incidence of AKI in Latin America (LA). Furthermore, most reports are not available in mainstream journals and are therefore not readily accessible with the usual search strategies. Lastly, the COVID-19 pandemic would have modified reports, at least by publication bias, but usual AKI data are still required[8].

To contribute to this information gap, we ought to perform a systematic review of the literature and meta-analysis of the epidemiology of AKI in Mexico. We aim to determine epidemiologic data on AKI and Kidney Replacement Therapy (KRT) in one of the most populated countries of LA that would contribute to general knowledge in this matter.

## Materials and methods

### Protocol and registration

The review was conducted based on the items outlined in the PRISMA [9] and MOOSE [10] statements. The protocol of this review is registered with the International Prospective Register of Systematic Reviews (PROSPERO) with ID CRD42022362196 [11].

### Criteria for including studies

Studies were considered eligible for inclusion if they were performed in Mexico. Articles with both pediatric and adult patients admitted to general ward facility, Emergency Room (ER), or Intensive Care Unit (ICU) were included. Eligible studies must have included data on the prevalence of adults with AKI, KRT or both. Articles were considered if published between January 1, 2000, and September 30, 2024, but reporting data prior to February 1, 2020. Observational and intervention studies with primary data using cross-sectional, case-control, and cohort (prospective and retrospective) designs were included.

Case reports, case series, narrative reviews, qualitative studies, editorials, commentaries, letters to editors, author replies, and animal studies were excluded. In addition, any study with fewer than 10 participants was not included, or if outcome measures were not available even after corresponding with the authors.

#### Types of outcomes

AKI has had various proposed definitions over time. The definition across studies varied from a combination of clinical variables (oliguria or anuria, urinary sediment), biochemical parameters (serum creatinine, biomarkers), to even International Classification of Diseases (World Health Organization) definitions. AKI may have been categorized by the intensity in stages, but, for the purposes of this review, AKI could have been either dichotomized as present or absent or as a worsening renal function. In the other hand, studies were also included if data on KRT in any modality was reported.

### Data sources and search strategy

The search terms were identified through a combination of keywords and MeSH terms related to acute kidney injury and Mexico, with iterative refinements based on the initial search results. The databases from MEDLINE (Pubmed interface), *Literatura Latinoamericana en Ciencias de la Salud* (LILIACS), EMBASE and Scientific Electronic Library Online (SciELO) were searched for eligible studies by JZG and AVR. As detailed in Supplementary Material A, the search terms were grouped into two categories: AKI and location (Mexico). Boolean operators "AND" and "OR" were used to combine the categories and terms within each category. A complete description of the search algorithm is detailed in Supplementary Material A. Results were limited to English, Spanish and Portuguese languages.

#### Searching other resources

We explored additional reports from the references of included records and previously published systematic review articles. Supplements, abstracts, and conference proceedings from Mexican and International Critical Care meetings were manually scanned. Personal collections of articles from the authors were also included.

An additional search was performed with the same strategy but comprising from January 1, 2020, up to September 30, 2024, to update the data from experience prior to COVID-19 reaching Latin America despite being published later (Supplementary material B).

### Data extraction

Two investigators (JJZ and AVR) independently screened reports for inclusion based on study titles and abstracts. They were assisted by a predefined electronic spreadsheet based on a series of *Yes* or *No* questions according to the above-mentioned selection criteria (Supplementary material C). The full text of articles deemed relevant during screening were retrieved and reviewed independently by them for inclusion. Disagreements between reviewers were solved by consensus or by a third reviewer (JSCI) if necessary.

Data extraction was comprehensive and included design (prospective *vs* retrospective cohort, nested case-control, case-cohort), sample size, AKI or KRT incidence or prevalence, type of patients, outcome(s) definitions, name of the hospital and city where the study took place, time of follow-up, etc. Data extraction was performed independently and in duplicate by two review authors into an electronic spreadsheet created for the purposes of this review. Disagreements were solved by discussion or by involving a third review author as arbiter.

The variables extracted were determined a priori based on our research objectives and the PICOS framework (Population, Intervention, Comparison, Outcome, and Study design). We prioritized extracting data on prevalence, incidence, mortality, and use of renal replacement therapy. When data were reported in different formats (e.g. prevalence, cumulative incidence), we standardized them to prevalence for the meta-analysis. Further details about the data extraction process and criteria can be found in Supplementary Material C.

### Risk of bias assessment

The risk of bias in the studies was independently carried out by two reviewers (JJZ and AVR) using an adapted version of the Critical Appraisal Skills Programme (CASP) tool [12] and STROBE statement [13]. A grading was assigned to various components of the study, including the pertinence of the study design for the research question, the risk of selection bias, classification bias, and outcome assessment. Reviewers were assisted by a set of questions or hints in each component (Supplementary material D). Grades were assigned for risk of bias as high, uncertain/not applicable, or low, and an overall judgment of bias was performed. Discrepancies were resolved by consensus or settled by a third reviewer (JSCI). A publication bias analysis was performed using the LFK index and Doi plot as recommended [14].

### Statistical analysis

We recalculated all the frequency estimates of any AKI or KRT occurrence if adequate data were provided by the authors. The 95% confidence intervals (95% CI) of the frequency were computed by the Wilson score method without continuity correction [15]. We performed a random-effects meta-analysis for clinically and methodologically comparable studies to estimate the frequency of AKI and KRT. We calculated pooled estimates stratified by age group (pediatrics and adults), year of publication (2000–2015 and 2016–2024), data collection (retrospective and prospective), hospital setting (ER, hospital ward, and ICU), and type of AKI definition (guideline or other). The heterogeneity between studies was assessed by Ι2 statistics. Statistical analysis was undertaken using STATA 16 (Stata Corp, College Station, TX).

## Results

**Figure 1.**
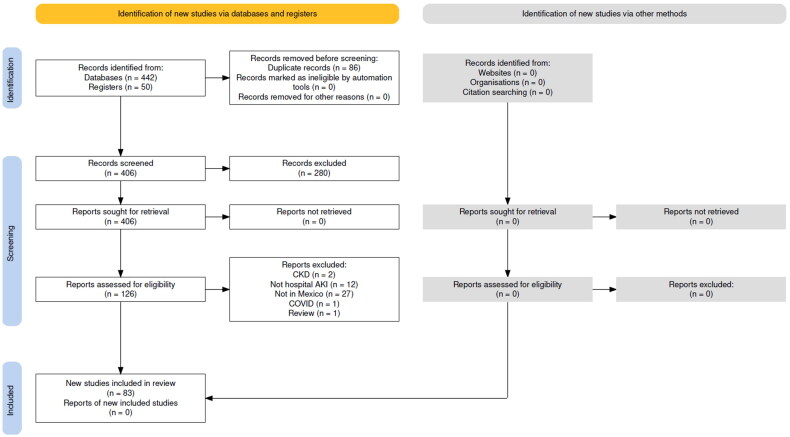
Study flow diagram.

After duplicate citations were removed, we screened 242 citations by title and abstract and selected 110 studies for full-text review. Ultimately, 71 full-text articles describing the epidemiology of AKI were included (Figure 1) [16–86]. Three of the included papers offered double data [21,69,80]. The additional updating search acknowledged 164 records that were screened by title and abstract, 16 of them were assessed full-text, and 12 were added to the final analysis [87–98]. These added reports are also described in Figure 1.

### Risk of bias assessment

**Figure 2.**
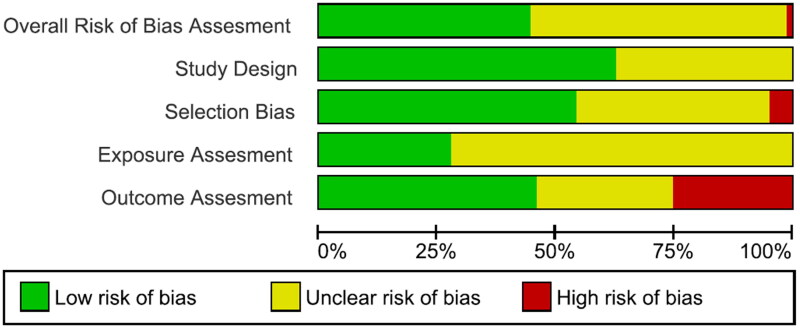
Risk of bias graph: review authors’ judgements about each risk of bias item presented as percentages across all included studies.

**Figure 3.**
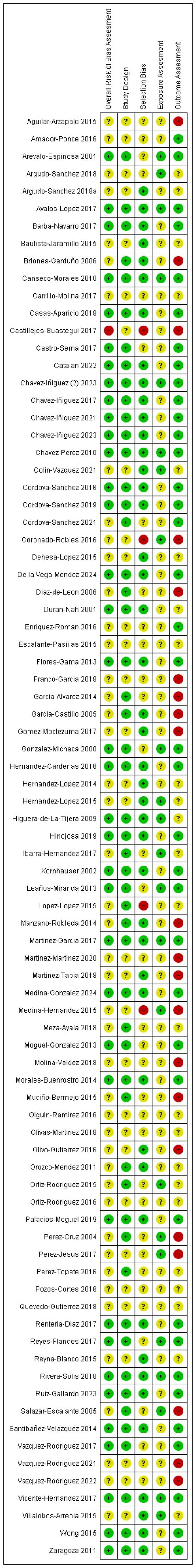
Risk of bias summary: review authors’ judgements about each risk of bias item for each included study.

Outcome assessment bias was found to be high in 25% of the studies. Only 42% of the papers were graded with an overall low risk of bias and 55% of them had an unclear risk of bias. The risk of bias graph and risk of bias summary are presented in Figures 2 and 3, respectively. A more detailed description of the author’s judgments can be found in Supplementary Material E.

### Acute kidney injury

**Figure 4.**
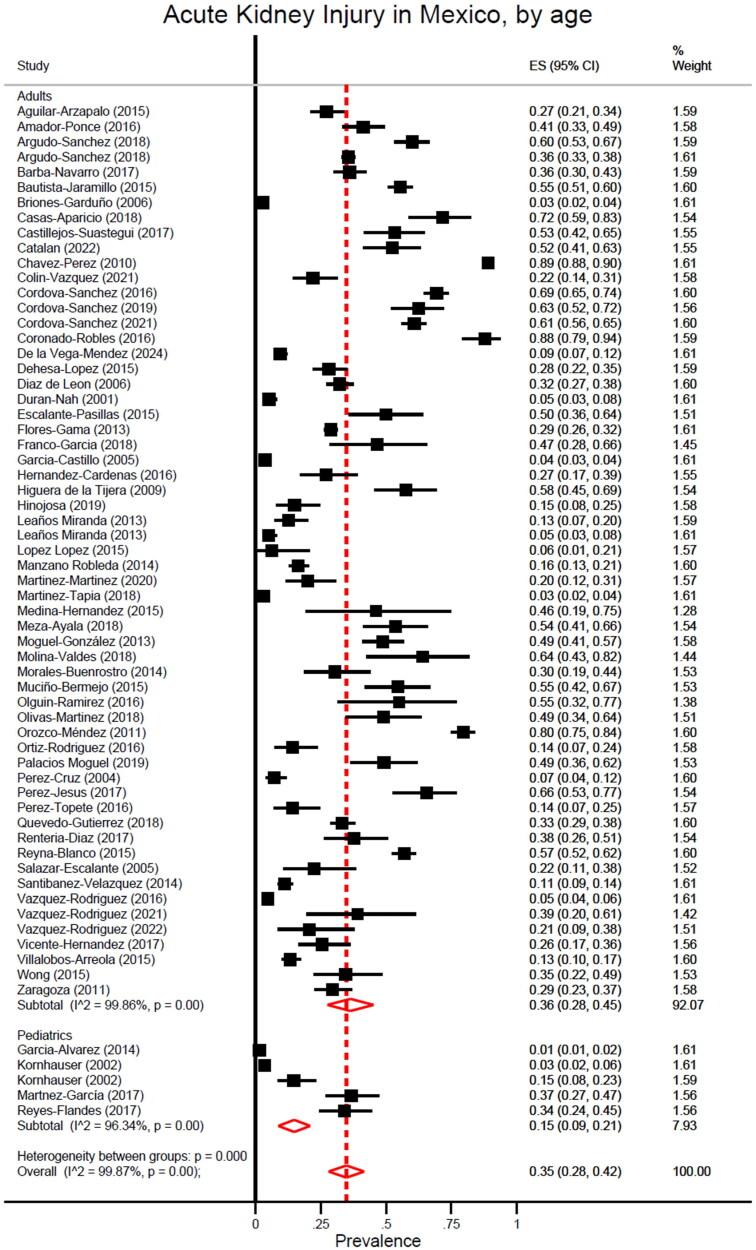
AKI by age subgroup.

In total, 72 reports presented data either in epidemiology or mortality rates for AKI in Mexico. A total of 69 of them contained data in epidemiology, merging a total of 35,918 patients to be analyzed. An overview of studies contributing to AKI information is available in Supplementary material F. Total percentage of AKI in Mexico was calculated at 35% (95% CI, 28–42). AKI in adults was calculated at 36% (95% CI, 28–45) and 15% (95% CI, 9–21) among pediatric patients. A global Ι2 was calculated in 99.87% (*p* < 0.01) with *p* < 0.01 for heterogeneity between age groups (Figure 4).

**Figure 5.**
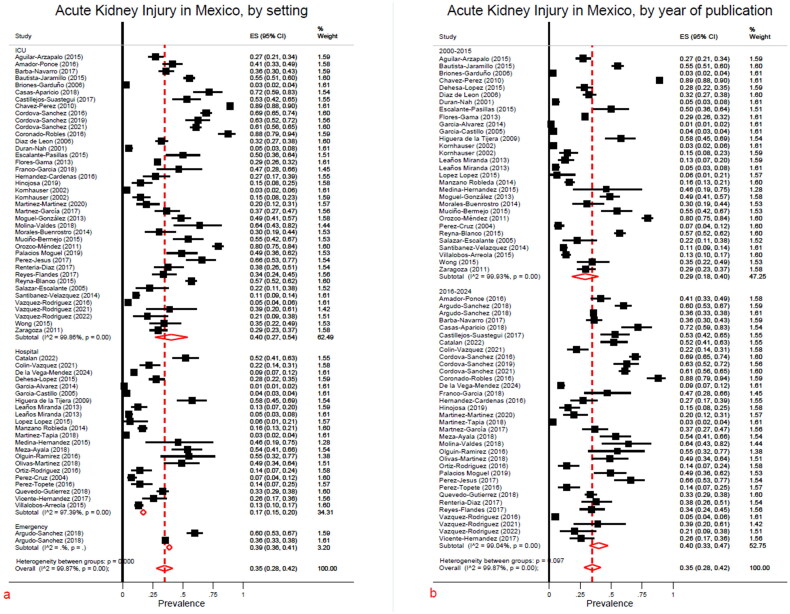
a) AKI by hospital setting, b) AKI by year of publication.

A subgroup analysis for the hospital setting was performed (Figure 5(a)). Studies were stratified by location such as ICU, hospital ward or ER. AKI frequency was reported in 40% (95% CI, 27–54), 17% (95% CI, 15–20), and 39% (95% CI, 36–41), respectively. In the same manner, heterogeneity between groups was reported as high. Studies were also divided by publication years, those issued between 2000 and 2015, and those performed between 2016 and 2024 (Figure 5(b)). A clear tendency to an increase in percentage reported was detected, from 29% (95% CI, 18–40) in the first period *vs* 40% (95% CI, 33–47) in the latter. Again, high heterogeneity was measured. An additional stratification analysis was performed using a type of definition, the prevalence of AKI was reported in 40% (95% CI, 26–54) of those papers using an international guideline, and 23% (95% CI, 21–26) when it was not specified or using a particular definition (Supplementary material G–a).

#### Mortality in acute kidney injury in Mexico

**Figure 6.**
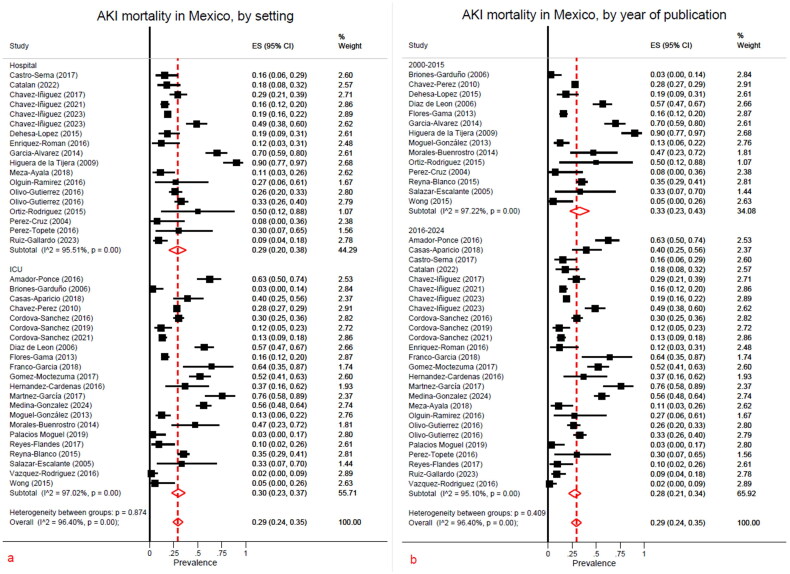
a) AKI mortality hospital setting, b) AKI by year of publication.

A sum of 41 studies presented data for mortality, with a total of 8,778 AKI patients. No studies on pediatric patients reported mortality. Mortality was calculated for AKI patients in Mexico in 29% (95% CI, 24–35). An overall Ι2 was estimated in 96.40% (*p* < 0.01). Subgroup analysis included hospital setting (Figure 6(a)) and date of publication (Figure 6(b)). Hospital ward AKI patients had a mortality of 29% (95% CI, 20–38) and in ICU 30% (95% CI, 23–37). A decrease in mortality rate was observed across year of publication, from a 33% (95% CI, 23–43) reported for those papers published between 2000 and 2015, to 28% (95% CI, 21–34) in studies from 2016 to 2024. In this case, heterogeneity between groups reported a *p* = 0.409.

## Kidney replacement therapy

Forty-one reports included information on KRT. Thirty-four studies were included in data analysis for KRT frequency in Mexico, with 17,786 patients integrated. Yet again, no pediatric papers that registered KRT were found. A description of the studies of KRT in Mexico can be analyzed in Supplementary Material H. An overall KRT rate was reported at 7% (95% CI, 6–9), and an overall Ι2 was estimated at 97.71% (*p* < 0.01). Hospital ward AKI patients reported KRT in 12% (95% CI, 6–17) and in ICU 6% (95% CI, 4–8). A *p* = 0.063 for heterogeneity between groups (Supplementary Material I – Figure A). No significant difference was found in the use of KRT in AKI across years (Supplementary Material I – Figure B).

### Mortality in kidney replacement therapy in Mexico

Only 20 reports provided data for KRT mortality in Mexico, a total of 1,846 patients were combined. All-cause mortality for AKI requiring KRT was estimated at 49% (95% CI, 42–56), with an Ι2 estimated at 81.46% (*p* < 0.01). Hospital ward patients that required KRT had a mortality rate of 36% (95% CI, 25–46) and 53% (95% CI, 45–61) in ICU. A *p* = 0.010 for heterogeneity between groups was calculated (Supplementary material J–Figure A). A small percentage decrease was calculated for studies after 2016 from 44% (95% CI, 36–53) as opposed to 56% (95% CI, 45–67) for those published in 2015 and earlier. A *p* = 0.385 for heterogeneity between groups was estimated (Supplementary material J – Figure B).

#### Quantitative assessment of bias (publication bias)

**Figure 7.**
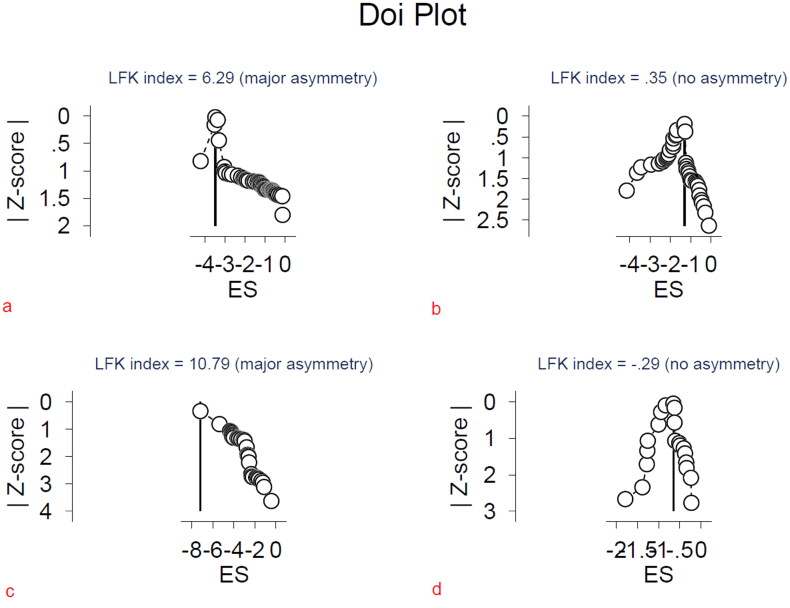
Doi Plot for: a) AKI, b) AKI mortality, c) KRT and d) KRT mortality.

Publication bias was evaluated by LFK index and Doi Plot as recommended for proportion meta-analysis [14,99,100]. The closer the value of the LFK index to zero, the more symmetrical the Doi Plot would be, zero represents complete symmetry. Major asymmetry was found in AKI and KRT frequencies, but not in AKI mortality or KRT mortality databases (Figure 7).

## Discussion

### Main findings

AKI frequency was reported in 36% of hospitalized patients in Mexico from 35,918 records included in the analysis. In ICU, hospital ward and ER, AKI rate was reported in 40%, 17% and 39% respectively. A clear tendency to an increase in percentage reported was observed, from 29% in 2000–2015 to 40% in 2015–2024. All-cause mortality was assessed in 29% of 8,778 AKI patients. Hospital ward AKI patients had a mortality of 29% and in ICU 30%. A decrease in mortality rate was observed across the year of publication, from 33% reported between 2000 and 2015, to 28% from 2016 to 2024.

After a systematic search, a total of 83 studies were ultimately included in the analysis. Some of them offered data on AKI, KRT, mortality or all of them. Unfortunately, most of the papers were of a low methodological quality or were published only as reports or abstracts. High risk of bias was common among the studies and publication bias is apparent as most reports are not available in mainstream journals and are, therefore, not readily accessible with the usual search strategies.

On 17,786 patients, KRT rate was reported in 7% of AKI patients, 12% of patients in general ward and 6% in ICU. From those who received KRT, a total of 1,846 patients, mortality for AKI requiring KRT was estimated to be 49%. Hospital ward patients that required KRT had a mortality rate of 36% and 53% in ICU; 44% for studies published after 2016 opposed to 56% for those issued in 2015 or earlier.

### Comparison with worldwide epidemiology

According to Melo et al. AKI incidence varied from 0.5% to 65% in developing countries. Sepsis and septic shock were the most common causes of AKI in both developed and developing countries. Pooled AKI incidence estimates for developed and developing countries in the meta-analysis including 9,174 patients was 35.1% (95% CI, 28.4–41.9). There was a non-significant tendency toward a greater incidence in developed countries, that disappeared when only prospective studies were analyzed. But developed countries had a 10-fold greater sample size than developing countries [101]. In developing countries, 48% of the 21 studies included in their analysis with available data showed that the frequency of KRT use was higher than 30% in critically ill. Reported ICU mortality in AKI patients was greater in developing country studies, more than 60% mortality rate was reported in 14 of 25 studies from developing countries [101].

A previous worldwide AKI incidence meta-analysis performed by Susantitaphong et al. in 2013 reported a pooled rate of AKI of 10.7% (95% CI, 9.6–11.9). When they restricted to the 154 studies that used a KDIGO-equivalent AKI definition, the pooled rate of AKI was 23.2% (95% CI, 21.0–25.7). Lower pooled AKI rates were observed in studies using administrative codes, which is a common practice in Mexico. In the same report, the pooled rate of dialysis requirement was 2.3%. As expected, the highest pooled AKI rate was observed in critical care settings, reaching up to 31.7% (95% CI, 28.6–35.0). When examined according to geographic regions, the pooled rate of AKI in South America (where Mexico was included) was 29.6%, higher compared with higher income countries. Our number reports a higher prevalence in hospital wards and critically ill patients. Contrary to what we found in this meta-analysis; the rate of AKI declined over the span of 8 years [5]. In other hand, the pooled AKI-associated all-cause mortality rate was 23.0% (95% CI, 21.3–24.8), 23.9% in adults (95% CI, 22.1–25.7) and 13.8% in children (95% CI, 8.8–21.0). We did not find a pediatric report in Mexican hospitals that reported mortality. Similarly to what we described; they showed that the AKI-associated mortality rate declined over the period of 8 year [5].

Two years later, Mehta et al. updated the previously mentioned work [6]. The search extended up to August 31, 2014. They added 266 papers that used KDIGO-equivalent AKI definitions, increasing sample size from 49 million individuals to more than 77 million individuals. Preliminary analysis of 4502158 patients showed that 21% of hospital admissions were affected by AKI [6]. The overall percentage of patients with AKI who needed dialysis was described as small (2% of hospital admissions; 11% of all AKI) but very similar to the pooled prevalence of KRT that we reported. The overall pooled mortality was 21% probably due to the predominance of patients with the more severe KDIGO stage 3 or those who require dialysis in agreement with our information. They did not include in their meta-analysis 33 studies from LA with 7023 patients because the report did not fit the KDIGO definition with a mean sample size 229 (range 13–879). Incidence of AKI in these papers was 7–12% in ICU admissions and 102 per 1 million population, pediatric admissions suffered 7% of AKI with a mortality of 43% [6].

A worldwide cohort published by Hoste et al. included information from few Mexican ICUs. They found an occurrence rate of maximum KDIGO stage 1 in 331 patients (18.4%; 95% CI, 16.7–20.2), KDIGO stage 2 in 161 patients (8.9%; 95% CI, 7.7–10.3), and KDIGO stage 3 in 540 patients (30.0%; 95% CI, 27.9–32.1). As expected, there was a stepwise increase in mortality with increasing AKI severity, even after adjustment for other variables that may explain mortality [102]. They also found a significant difference in the occurrence of AKI and in mortality for patients with AKI between continents and world. However, adjusted rates for AKI and mortality were quite similar across different continents. When countries were grouped according to income, proportion of GDP spend for health expenditure, or latitude, the rates of AKI and mortality associated with AKI were also similar [102]. During the whole 1-week study period, a total of 243 patients were treated with KRT; 13.5% of all patients (95% CI, 12.0–15.1), and 23.5% of AKI patients (95% CI, 21.1–26.2) [102]. Expected mortality was lower than observed (actual) mortality, although the difference was not statistically significant in Central America, where Mexico was included.

### Previously reported incidence in Latin America and Mexico

AKI in Latin America has been understudied, but it is believed to have a bimodal pattern. In urban areas, AKI characteristics are like those found in high-income countries; a predominantly hospital-acquired disease affecting mostly older, critically ill multiorgan failure patients. On the other hand, in remote and poor areas, AKI affects mainly young and previously healthy individuals, with a profile strongly influenced by environmental conditions and socioeconomic and cultural status [3]. It is estimated that about 29.6% of hospitalized adult or pediatric patients will develop AKI in Latin America [3, 7, 103]. Of the estimated 1.7 million deaths from AKI per year, 80% of them occur in low-medium income countries, and 88% of them in the first or second level of care [7]. The incidence of AKI has been estimated between 322 and 522 cases per 100,000 population, relating to a mortality rate of 20 to 60% [3].

Mexico lacks a national AKI registry, and therefore, inaccuracy is expected on the reported epidemiology of the disease. An urban tertiary level of care registry suggests a that 64% of ICU patients will develop AKI, with sepsis as the most common etiology in 70% of the cases [4]. In total, 30% of patients were reported requiring some type of KRT and mortality around 22.8% [4]. Unfortunately, subjects who survive an episode of AKI are rarely followed up, with limited or null data on long term outcomes [4].

### Special situations

In a previous review published by Chavez-Iñiguez in 2018, it was described that they found publications on AKI only from 10 LA countries; and merely 2.2% corresponding to pediatric population. The most frequent causes of AKI were critical condition, cardiac disease and sepsis representing 72% of the reports. Other reported causes were nephrotoxins, infectious diseases such as influenza, dengue, toxoplasmosis, leptospirosis and Human Immunodeficiency Virus (HIV) infection, liver disease, cancer, prostatic resection and obstetric-related AKI [7]. As found in our search, it seems that publication bias is common in LA, with a high frequency of monovalent ICUs and large academic centers producing publications. More than half of the reports included in our review were performed attending a single type of patient, the stated causes and incidence might have been influenced by this.

In this context, Tariq et al. reported in 2020 an analysis of 18,474 patients with cirrhosis, 5,648 developed AKI, with a pooled incidence of 29% (95% CI, 28–30) [104]. In-hospital mortality was assessed in eight studies. The rate of mortality among AKI patients was 34.6% vs 9.7% compared to those without AKI (OR 6.72; 95% CI, 3.47–13; *p* < 0.0001) [104]. Colin-Vazquez et al. found in 86 Liver Transplantation (LT) patients, that 45 (52%) developed AKI in the following 30 days after LT. Nine patients (10.4%) required KRT. After a follow-up of 30 days, 11.6% died [88].

### Incidence of AKI during COVID-19 pandemic

We decided to limit our research to December 31, 2019. The first case of COVID-19 in Mexico was reported in February 2020, we believe that avoiding possible bias of including COVID patients was the way to go. However, the 2020 pandemic deserves a special mention. In 2021, Casas-Aparicio et al. published a great retrospective study of 99 COVID-19 patients in Mexico, exploring the relationship of AKI and mortality [105]. Fifty-eight patients developed AKI (58.6%) and 41 individuals did not develop AKI. Of those, 12 had AKI stage 1 (21.1%); 16 had AKI stage 2 (28.1%); and 29 had AKI stage 3 (50.9%). On the survival analysis by day 30 of follow-up, mortality was significantly higher in the AKI group than the non-AKI group (65.5% vs. 14.6%, *p* = 0.001). A total of 11 patients (22.4%) required KRT. Of those, five used continuous KRT, three used intermittent hemodialysis, and three used prolonged intermittent KRT [105].

It is important to contextualize the relevance of AKI mortality, which is higher when compared to other more common pathologies. The mortality for AKI was 31% in our study, compared with 6.3% in patients admitted for myocardial infarction in Latin America and the Caribbean [106], 9.1% in ischemic stroke [107] or 30% observed in pneumonia patients >75 years [108].

### Strengths and limitations

Data for epidemiological studies in LA are scarce. To our best knowledge, this is the largest and most recent systematic review and meta-analysis of AKI epidemiology in Mexico. We performed search as broad as possible, since widespread of AKI databases and/or registries is limited. Additionally, manual assessment of national critical care and nephrology meetings reports and addition of authors’ personal library is believed to be a strength. The period of research after the publication of RIFLE, AKIN or KDIGO consensus, rendered papers that supposedly had a homologized way to evaluate AKI.

Our limitations are that we decided not to include pandemic data, as AKI epidemiology may have been modified during this period, and reports were published in an uncontrolled manner in our country. Furthermore, methodological quality of epidemiological reports in our country is low, as demonstrated by the risk of bias assessment with most of the papers evaluated as having a high or unclear risk of bias. Additionally, publication bias is evident, we do not believe that the sensitivity analysis by subgroups solved this issue completely. The preprint of this manuscript can be found online [109].

## Conclusions

This systematic review and meta-analysis showed that AKI is a common disease in our country, in ER, ICU and hospital ward, are still a main public health problem that needs to be addressed at every level of care. Data for epidemiological studies in Latin America is scarce and reports are usually published in an uncontrolled manner with a low methodological quality in our country. AKI epidemiology may have been modified during COVID-19 pandemic, but efforts should be made to reinitiate AKI research and control in Mexico and LA.

## Supplementary Material

SM G.pdf

SM J.pdf

Supplementary material 09_12_24.docx

PRISMA_2020_checklist.pdf

MOOSE.pdf

SM I.pdf

## Data Availability

The data that support the findings of this study are available from the corresponding author AVR and you can contact drarmandovazquez@hotmail.com

## Abbreviations

AKI: Acute Kidney Injury
KDIGO: Kidney Disease Improving Global Outcomes
LA: Latin America
KRT: Kidney Replacement Therapy
PROSPERO: Prospective Register of Systematic Reviews
ER: Emergency Room
ICU: intensive care unit
LILIACS: Literatura Latinoamericana en Ciencias de la Salud
SciELO: Scientific Electronic Library Online
HIV: Human Immunodeficiency Virus
LT: Liver Transplantation
