# Supplementary figures and images for: Prevalence of acute kidney injury in Mexico; a systematic review and meta-analysis of pre-pandemic reports
Source: Ren Fail. 2025 Jan 30;47(1):2449573. doi: 10.1080/0886022X.2024.2449573 (PMC11784032; doi:10.1080/0886022X.2024.2449573)

# Acute Kidney Injury in Mexico, by AKI definition

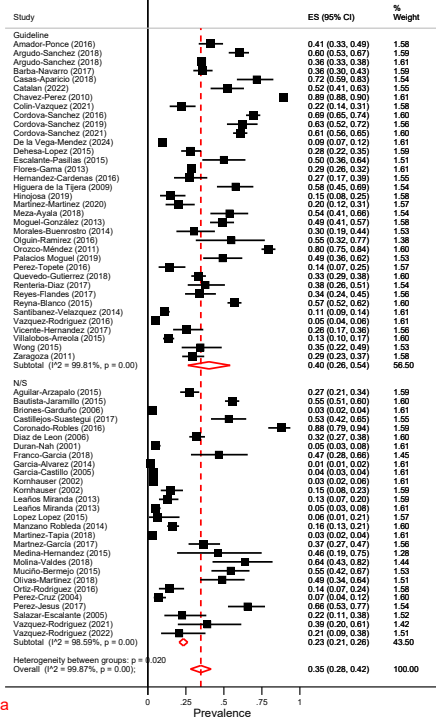

# AKI mortality in Mexico, by AKI definition

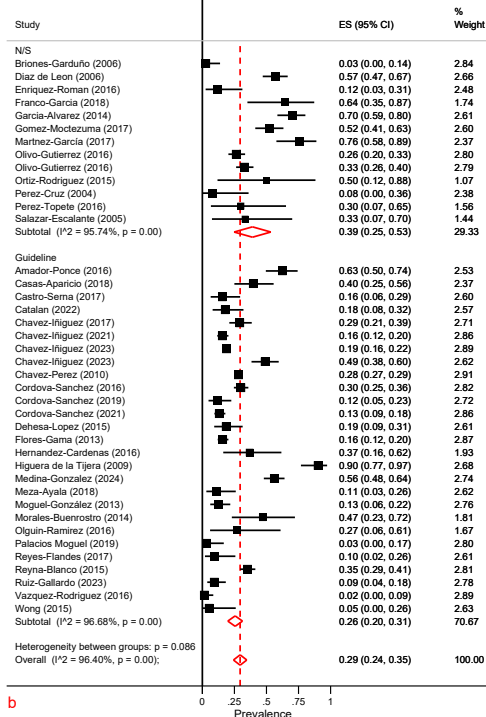

Supplement: SM G.pdf [file IRNF_A_2449573_SM4701.pdf]

## RRT mortality in Mexico, by setting

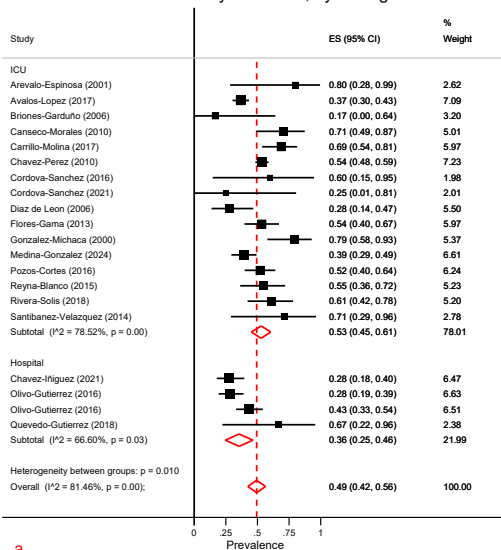

## RRT mortality in Mexico, by year of publication

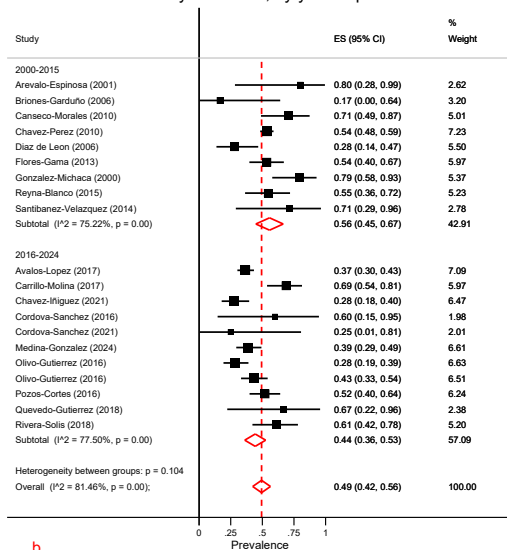

Supplement: SM J.pdf [file IRNF_A_2449573_SM4700.pdf]

## RRT in Mexico, by setting

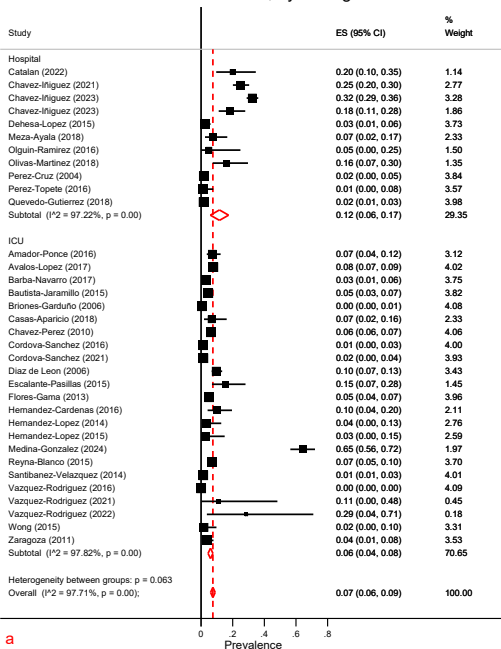

a

## RRT in Mexico, by year of publication

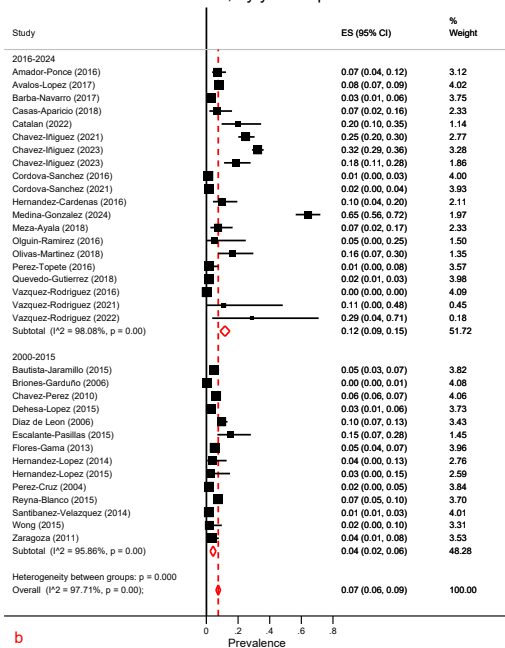

b

Supplement: SM I.pdf [file IRNF_A_2449573_SM4696.pdf]
