# Supplementary material for: Prevalence of acute kidney injury in Mexico; a systematic review and meta-analysis of pre-pandemic reports
Source: Ren Fail. 2025 Jan 30;47(1):2449573. doi: 10.1080/0886022X.2024.2449573 (PMC11784032; doi:10.1080/0886022X.2024.2449573)

Supplementary material A – Search strategy

| Dec 31  2019 | Medline (Pubmed) |  |
| --- | --- | --- |
| Number | Algorithm | Results |
| #1 | ("acute kidney injury"[MeSH Terms]) AND (2000/1/1:2019/12/31[pdat]) | 26044 |
| #2 | ("mexico"[MeSH Terms] OR "mexico"[All Fields] OR "mexico s"[All Fields] OR "mexicos"[All Fields])  AND (2000/1/1:2019/12/31[pdat]) | 126099 |
| #3 | #1 AND #2 | 156 |

| Dec 31  2019 | SCOPUS |  |
| --- | --- | --- |
| Number | Algorithm | Results |
| #1 | TITLE-ABS-KEY ( acute AND kidney AND injury ) AND PUBYEAR > 1999 AND PUBYEAR <  2020 AND PUBYEAR > 1999 AND PUBYEAR < 2020 | 42848 |
| #2 | TITLE-ABS-KEY ( mexico ) AND PUBYEAR > 1999 AND PUBYEAR < 2020 AND PUBYEAR >  1999 AND PUBYEAR < 2020 | 146774 |
| #3 | #1 AND #2 | 55 |

| Oct. 23 | LILACS |  |
| --- | --- | --- |
| Number | Algorithm | Results |
| #1 | Falla renal aguda or Daño renal agudo or Injuria renal aguda or insuficiencia renal aguda [Palabras] | 1176 |
| #2 | mexico [Palabras] | 18130 |
| #3 | #1 AND #2 | 7 |

| Oct. 23 | EMBASE |  |
| --- | --- | --- |
| Number | Algorithm | Results |
| #1 | Acute Kidney Injury/ limit to yr="2000 - 2019" | 25176 |
| #2 | exp Mexico/ | 42767 |
| #3 | #1 AND #2 | 13 |

Supplementary material B – Additional update Search strategy

| Jan 1, 2020, Sept 30, 2024 | Medline (Pubmed) |  |
| --- | --- | --- |
| Number | Algorithm | Results |
| #1 | ("acute kidney injury"[MeSH Terms]) AND ((ffrft[Filter]) AND (2020/1/1:2024/9/30[pdat])) | 7182 |
| #2 | ("mexico"[MeSH Terms] OR "mexico"[All Fields] OR "mexico s"[All Fields] OR "mexicos"[All Fields]) AND ((ffrft[Filter]) AND (2020/1/1:2024/9/30[pdat])) | 126099 |
| #3 | #1 AND #2 | 98 |

| Jan 1, 2020, Sept 30, 2024 | SCOPUS |  |
| --- | --- | --- |
| Number | Algorithm | Results |
| #1 | TITLE-ABS-KEY ( acute AND kidney AND injury ) AND PUBYEAR > 1999 AND PUBYEAR <  2020 AND PUBYEAR > 1999 AND PUBYEAR < 2020 | 35281 |
| #2 | TITLE-ABS-KEY ( mexico ) AND PUBYEAR > 1999 AND PUBYEAR < 2020 AND PUBYEAR >  1999 AND PUBYEAR < 2020 | 58259 |
| #3 | #1 AND #2 | 62 |

| 2020 - 2024 | LILACS |  |
| --- | --- | --- |
| Number | Algorithm | Results |
| #1 | (falla renal aguda) OR (daño renal agudo) OR (injuria renal aguda) OR (insuficiencia renal aguda) AND (year_cluster:[2020 TO 2024]) AND (instance:"lilacsplus") | 1178 |
| #2 | (mexico) AND (year_cluster:[2020 TO 2024]) AND (instance:"lilacsplus") | 15217 |
| #3 | #1 AND #2 | 55 |

| 2020 - 2024 | TOTAL | 215 |
| --- | --- | --- |
| Number | Search | Results |
| #1 | Screened for pre-COVID information | 23 |
| #2 | After removing duplicates | 16 |
| #3 | Included for full text review | 12 |

Supplementary material C – Inclusion/Exclusion form

**Study Eligibility Form ^1^**

| Reviewer | CECT |  | JMLT |
| --- | --- | --- | --- |
| Article Title |  | | |
| Reference #: | Author: | Journal: | Year: |

| Population |  |  |
| --- | --- | --- |
| AKI or KRT Patients^2^ | Yes | No |
| Adult or pediatric patients | Yes | No |
| Located in Mexico | Yes | No |

| Design |  |  |
| --- | --- | --- |
| More than 10 participants | Yes | No |
| Cross-sectional, case–control, and cohort (prospective and retrospective)  designs. | Yes | No |

| Outcome reported |  |  |
| --- | --- | --- |
| Included data on the prevalence or incidence | Yes | No |

| Timing and setting |  |  |
| --- | --- | --- |
| Intensive Care (or similar) or General Ward Facility | Yes | No |

Study inclusion:

All the answers are YES = INCLUDE Any answer is NO = EXCLUDE

1. If any response to the above questions is unclear, mark YES.
2. Any type of predictor, including but not limited to clinical characteristics, laboratory values, test results and any other clinical event.

Supplementary material D – Risk of bias assessment tool

| Domain | Questions or Hints to classify | Risk of Bias |
| --- | --- | --- |
| Study design | - Were appropriate data sources used, that is, cohort, RCT or nested   case-control study data?   - Were all inclusions and exclusions of participants appropriate? - Were participants enrolled at a similar state of health, or were predictors considered to account for differences? - Assign a “low risk” grading if the research question was on the risk/prognostic factors for acute kidney injury for case-control and cohort studies (and not cross-sectional studies). - Assign a “low risk” grading if cross-sectional and cohort studies   studied only the epidemiology of acute kidney injury. | High  Moderate  Low Unclear |
| Selection bias | - Were the controls representative of the defined population   (geographically and/or temporally)?   - Was there something special about the controls? - Are they matched, population based or randomly selected? - Were there enough controls selected? | High  Moderate  Low Unclear |
| Classification bias | - Was the exposure clearly defined and accurately measured? - Did the authors use subjective or objective measurements? - Do the measures truly reflect what they are supposed to measure (have they been validated)? - Were the measurement methods similar in the cases and controls? - Did the study incorporate blinding where feasible is the temporal relation correct (does the exposure of interest precede the outcome)? | High  Moderate  Low Unclear |
| Outcome assessment | - Was a pre-specified outcome definition used? - Were predictors excluded from the outcome definition? - Are the design and methods of this study sufficiently flawed to make the results unreliable? - Was the outcome defined and determined in a similar way for all participants? - Was the outcome determined without knowledge of predictor information? | High  Moderate  Low Unclear |
| Overall Risk of Bias | High  Moderate  Low Unclear | |

Supplementary material E – Risk of Bias Summary

| **Author** |  | **Overall risk of bias assessment** | **Components of risk of bias assessment** | | | |
| --- | --- | --- | --- | --- | --- | --- |
|  | Year |  | **Study design** | **Selection bias** | **Exposure assessment** | **Outcome assessment** |
| Aguilar-Arzapalo (51) | 2015 | Moderate | Moderate | Moderate | Not Applicable | High |
| Amador-Ponce (50) | 2016 | Moderate | Moderate | Moderate | Not Applicable | Low |
| Arevalo-Espinosa (55) | 2001 | Low | Low | Moderate | Low | Low |
| Argudo-Sanchez (48,49) | 2018 | Moderate | Moderate | Moderate | Low | Moderate |
| Argudo-Sanchez (48,49) | 2018 | Moderate | Moderate | Low | Not Applicable | Moderate |
| Avalos-Lopez (86) | 2017 | Low | Low | Low | Low | Low |
| Barba-Navarro (85) | 2017 | Low | Low | Low | Not Applicable | Low |
| Bautista-Jaramillo (47) | 2015 | Moderate | Moderate | Low | Not Applicable | Moderate |
| Briones-Garduño (84) | 2006 | Moderate | Low | Low | Moderate | High |
| Canseco-Morales (54) | 2010 | Low | Low | Low | Low | Low |
| Carrillo-Molina (46) | 2017 | Moderate | Moderate | Moderate | Not Applicable | Moderate |
| Casas-Aparicio (83) | 2018 | Low | Low | Low | Not Applicable | Low |
| Castillejos-Suastegui (45) | 2017 | High | Moderate | High | Not Applicable | High |
| Castro-Serna (65) | 2017 | Low | Low | Moderate | Not Applicable | Low |
| Chavez-Iñiguez (53) | 2017 | Low | Low | Low | Not Applicable | Low |
| Chavez-Perez (44) | 2010 | Low | Low | Low | Low | Low |
| Cordova-Sanchez (82) | 2016 | Low | Low | Low | Not Applicable | Low |
| Cordova-Sanchez (43) | 2019 | Low | Low | Low | Not Applicable | Low |
| Coronado-Robles (42) | 2016 | Moderate | Moderate | High | Low | High |
| Dehesa-Lopez (41) | 2015 | Moderate | Moderate | Low | Not Applicable | Moderate |
| Diaz de Leon (52) | 2006 | Moderate | Low | Moderate | Not Applicable | High |
| Duran-Nah (40) | 2001 | Low | Low | Low | Not Applicable | Moderate |
| Enriquez-Roman (39) | 2016 | Moderate | Moderate | Moderate | Not Applicable | Low |
| Escalante-Pasillas (38) | 2015 | Moderate | Moderate | Moderate | Not Applicable | Moderate |
| Flores-Gama (70) | 2013 | Low | Low | Low | Not Applicable | Low |
| Franco-Garcia (37) | 2018 | Moderate | Moderate | Moderate | Not Applicable | High |
| Garcia-Alvarez (36) | 2014 | Moderate | Low | Moderate | Not Applicable | High |
| Garcia-Castillo (35) | 2005 | Moderate | Low | Low | Not Applicable | High |
| Gomez-Moctezuma (34) | 2017 | Moderate | Moderate | Low | Not Applicable | High |
| Gonzalez-Michaca (33) | 2000 | Low | Low | Moderate | Low | Low |
| Hernandez-Cardenas (32) | 2016 | Low | Low | Low | Not Applicable | Low |
| Hernandez-Lopez (31) | 2014 | Moderate | Moderate | Low | Not Applicable | Moderate |
| Hernandez-Lopez (30) | 2015 | Moderate | Moderate | Low | Low | Moderate |
| Higuera de la Tijera (64) | 2009 | Low | Low | Low | Low | Moderate |
| Hinojosa (81) | 2019 | Low | Low | Low | Not Applicable | Low |
| Ibarra-Hernandez (69) | 2017 | Moderate | Low | Moderate | Low | Moderate |
| Kornhauser (80) | 2002 | Low | Low | Low | Not Applicable | Low |
| Leaños Miranda (79) | 2013 | Low | Low | Moderate | Low | Low |
| Lopez Lopez (78) | 2015 | Moderate | Low | High | Not Applicable | Moderate |
| Manzano-Robleda (56) | 2014 | Moderate | Low | Low | Not Applicable | High |
| Martinez-Tapia (28) | 2018 | Moderate | Moderate | Low | Not Applicable | High |
| Martinez-García (77) | 2017 | Low | Low | Low | Low | Low |
| Medina-Hernandez (27) | 2015 | Moderate | Moderate | High | Low | High |
| Meza-Ayala (57) | 2018 | Moderate | Low | Moderate | Not Applicable | Moderate |
| Moguel-González (76) | 2013 | Low | Low | Moderate | Not Applicable | Low |
| Molina-Valdes (26) | 2018 | Moderate | Moderate | Moderate | Not Applicable | High |
| Morales-Buenrostro (75) | 2014 | Low | Low | Low | Not Applicable | Low |
| Muciño-Bermejo (74) | 2015 | Moderate | Low | Moderate | Not Applicable | High |
| Olguin-Ramirez (23) | 2016 | Moderate | Moderate | Moderate | Not Applicable | Moderate |
| Olivas-Martinez (22) | 2018 | Moderate | Moderate | Moderate | Not Applicable | Moderate |
| Olivo-Gutierrez (21) | 2016 | Moderate | Moderate | Low | Not Applicable | High |
| Orozco-Méndez (58) | 2011 | Moderate | Low | Low | Moderate | Moderate |
| Ortiz-Rodriguez (25) | 2015 | Moderate | Low | Moderate | Low | Moderate |
| Ortiz-Rodriguez (24) | 2016 | Moderate | Moderate | Moderate | Not Applicable | Moderate |
| Palacios Moguel (59) | 2019 | Low | Low | Low | Not Applicable | Low |
| Perez-Cruz (16) | 2004 | Moderate | Low | Moderate | Low | High |
| Perez-Jesus (20) | 2017 | Moderate | Moderate | Moderate | Low | High |
| Perez-Topete (60) | 2016 | Moderate | Low | Moderate | Not Applicable | Moderate |
| Pozos-Cortes (19) | 2016 | Moderate | Moderate | Moderate | Not Applicable | Moderate |
| Quevedo-Gutierrez (18) | 2018 | Moderate | Moderate | Moderate | Not Applicable | Moderate |
| Renteria-Diaz (61) | 2017 | Low | Low | Low | Not Applicable | Low |
| Reyes-Flandes (73) | 2017 | Low | Low | Moderate | Low | Low |
| Reyna-Blanco (17) | 2015 | Moderate | Moderate | Low | Not Applicable | Moderate |
| Rivera-Solis (62) | 2018 | Low | Low | Low | Low | Low |
| Salazar-Escalante (63) | 2005 | Moderate | Low | Moderate | Low | High |
| Santibanez-Velazquez (72) | 2014 | Low | Low | Low | Moderate | Low |
| Vazquez-Rodriguez (71) | 2016 | Low | Low | Moderate | Not Applicable | Low |
| Vicente-Hernandez (66) | 2017 | Low | Low | Low | Low | Low |
| Villalobos-Arreola (29) | 2015 | Moderate | Moderate | Low | Low | Moderate |
| Wong (68) | 2015 | Low | Low | Low | Not Applicable | Low |
| Zaragoza (67) | 2011 | Low | Low | Low | Not Applicable | Low |
| Cordova-Sanchez (87) | 2021 | Moderate | Low | Moderate | Not applicable | Low |
| Catalan (88) | 2022 | Low | Low | Low | Not applicable | Low |
| Chavez-Iñiguez (89) | 2023 | Low | Low | Low | Not appliable | Low |
| Chavez-Iñiguez (90) | 2023 | Low | Low | Low | Low | Low |
| Chavez-Iñiguez (91) | 2021 | Low | Low | Low | Moderate | Low |
| Colin-Vazquez (92) | 2021 | Moderate | Moderate | Low | Low | Moderate |
| De la Vega-Mendez (93) | 2024 | Low | Low | Low | Not appliable | Low |
| Martinez-Martinez (94) | 2020 | Moderate | Moderate | Moderate | Not applicable | High |
| Medina-Gonzalez (95) | 2024 | Low | Low | Low | Not appliable | Low |
| Ruiz-Gallardo (96) | 2023 | Low | Low | Low | Not appliable | Low |
| Vazquez-Rodriguez (97) | 2022 | Moderate | Moderate | Moderate | Not applicable | High |
| Vazquez-Rodriguez (98) | 2021 | Moderate | Moderate | Moderate | Not applicable | High |

Supplementary material F. Summary of included studies in AKI information.

| **Author** | **Year** | **Setting** | **Study population (n)** | | | **Type of patients** | **Age of subjects** | **Outcome studied and assessment method** | | **Design** | **Main results Percentage** | **Overall risk of bias assessment** |
| --- | --- | --- | --- | --- | --- | --- | --- | --- | --- | --- | --- | --- |
|  |  |  | **AKI** | **Total** | **AKI**  **mortality** |  |  | **Outcome Definition** | **Method of outcome**  **assessment** |  |  |  |
| Aguilar-Arzapalo (51) | 2015 | ICU | 56 | 206 | NS | Mixed | Adults | NS | NS | Prospective | AKI 27.2% | **Moderate** |
| Amador-Ponce  (50) | 2016 | ICU | 64 | 155 | 40 | Mixed | Adults | AKIN | SCr or UO | Prospective | AKI 41.3% | **Moderate** |
| Argudo-Sanchez (,48,49) | 2018 | ER | 132 | 220 | NS | Mixed | Adults | KDIGO | SCr | Retrospective | AKI 59.9%, severe AKI 24.0% | **Moderate** |
| Argudo-Sanchez (48,49) | 2018 | ER | 498 | 1398 | NS | Mixed | Adults | KDIGO | SCr | Retrospective | AKI 35.6%, severe AKI 12.0% | **Moderate** |
| Barba-Navarro (85) | 2017 | ICU | 84 | 233 | NS | Cardiac surgery | Adults | KDIGO | SCr or UO | Prospective | AKI 36%, severe AKI 7% | **Low** |
| Bautista-Jaramillo (47) | 2015 | ICU | 249 | 449 | NS | Mixed | Adults | NS | NS | Retrospective | AKI 55.5% | **Moderate** |
| Briones-Garduño (84) | 2006 | ICU | 38 | 1272 | 1 | Obstetrics | Adults | Own | CrCl < 15  ml/min | Retrospective | AKI 3% | **Moderate** |
| Casas-Aparici o (83) | 2018 | ICU | 43 | 60 | 17 | Pneumonia by AH1N1 | Adults | KDIGO | SCr or UO | Retrospective | AKI 71.7%,  severe AKI 56.7% | **Low** |
| Castillejos-Suastegui (45) | 2017 | ICU | 41 | 77 | NS | Mixed | Adults | NS | NS | Retrospective | AKI 53.2% | **High** |
| Castro-Serna (65) | 2017 | Hospital | 45 | NS | 7 | Cirrhosis | Adults | KDIGO | SCr | Prospective | AKI Mortality 15.5%, 18.9% for severe AKI | **Low** |
| Chavez-Iñiguez (53) | 2017 | Hospital | 113 | NS | 33 | Mixed | Adults | AKIN | SCr | Prospective | Mortality 29% for  AKI, 39.7%  for severe AKI | **Low** |
| Chavez-Perez (44) | 2010 | ICU | 5188 | 5829 | 1378 | Mixed | Adults | RIFLE | SCr or UO | Prospective | AKI 83.9%,  severe AKI 73.5% | **Low** |
| Cordova-Sanchez (82) | 2016 | ICU | 270 | 389 | 81 | Cancer | Adults | KDIGO | SCr or UO | Retrospective | AKI 69.4%, severe AKI 43.4% Mort. 30% for AKI, 40.2% for severe AKI | **Low** |
| Cordova-Sanchez (43) | 2019 | ICU | 60 | 96 | 7 | Cancer | Adults | KDIGO | SCr or UO | Prospective | AKI 63%, severe AKI 34.4%. Mortality 12% | **Low** |
| Coronado-Robles (42) | 2016 | ICU | 79 | 90 | NS | Mixed | Adults | NS | NS | Prospective | AKI 88.0% | **Moderate** |
| Dehesa-Lopez (41) | 2015 | Hospital | 54 | 192 | 10 | Mixed | Adults | AKIN | SCr | Retrospective | AKI 28.1%,  severe AKI 13.6% | **Moderate** |
| Diaz de Leon  (52) | 2006 | ICU | 107 | 332 | 61 | Peritonitis | Adults | NS | NS | Retrospective | Severe AKI 32.2% | **Moderate** |
| Duran-Nah  (40) | 2001 | ICU | 16 | 306 | NS | Mixed | Adults | Own | (SCr) ⃰ 1.2 | Retrospective | AKI 5.2% | **Low** |
| Enriquez-Roman (39) | 2016 | Hospital | 25 | NS | 3 | Cancer | Adults | NS | NS | Retrospective | Mortality 12.0% | **Moderate** |
| Escalante-Pasillas (38) | 2015 | ICU | 26 | 52 | NS | Mixed | Adults | AKIN stage 1 | SCr | Prospective | AKI 50.0% | **Moderate** |
| Flores-Gama (70) | 2013 | ICU | 316 | 1096 | 50 | Cardiac surgery | Adults | AKIN | SCr | Retrospective | AKI 28.8%,  severe AKI 6.8% | **Low** |
| Franco-Garcia  (37) | 2018 | ICU | 14 | 30 | 9 | Mixed | Adults | NS | NS | Retrospective | AKI 46.0% | **Moderate** |
| Garcia-Alvarez  (36) | 2014 | Hospital | 75 | 5039 | NS | Mixed | Pediatrics | NS | NS | Retrospective | AKI 1.5% | **Moderate** |
| Garcia-Castillo (35) | 2005 | Hospital | 300 | 8098 | NS | Coronary syndrome | Adults | NS | NS | Prospective | AKI 3.7% | **Moderate** |
| Gomez-Moctezuma (34) | 2017 | ICU | 86 | NS | 45 | Mixed | Adults | NS | NS | Retrospective | Mortality  52.0% | **Moderate** |
| Hernandez-Cardenas (32) | 2016 | ICU | 19 | 70 | 7 | Pneumonia by influenza A H1N1 | Adults | AKIN | NS | Prospective | AKI 27.1%,  severe AKI 22.8% | **Low** |
| Higuera de la  Tijera (64) | 2009 | Hospital | 41 | 71 | 37 | Alcoholic hepatitis | Adults | AKIN stage 1 | SCr | Prospective | AKI 57.7% | **Low** |
| Hinojosa (81) | 2019 | ICU | 12 | 80 | NS | Aortic aneurism | Adults | KDIGO | NS | Retrospective | AKI 15% | **Low** |
| Kornhauser (80) | 2002 | ICU | 15 | 430 | NS | Mixed | Pediatrics | Own | SCr > 1.2 mg/dl or UO < 1 ml/kg/h | Prospective | AKI 3.5% | **Low** |
| Kornhauser (80) | 2002 | ICU | 15 | 102 | NS | Mixed | Pediatrics | Own | SCr > 1.2 mg/dl or UO < 1 ml/kg/h | Prospective | AKI 14.7% | **Low** |
| Leaños  Miranda (79) | 2013 | Hospital | 15 | 118 | NS | Obstetric | Adults | Own | SCr > 2.2  mg/dl | Prospective | AKI 12.7% | **Low** |
| Leaños  Miranda (79) | 2013 | Hospital | 13 | 261 | NS | Obstetric | Adults | Own | SCr > 2.2  mg/dl | Prospective | AKI 5% | **Low** |
| Lopez Lopez  (78) | 2015 | Hospital | 2 | 32 | NS | Percutaneous coronary  intervention | Adults | Own | (SCr) ⃰ 1.25 or  ↑ ≥ 0.5 mg/dl | Prospective | AKI 6.3% | **Moderate** |
| Manzano-Robleda (56) | 2014 | Hospital | 60 | 367 | NS | Acute diarrhea | Adults | NS | NS | Retrospective | AKI 16.3% | **Moderate** |
| Martinez-Tapia  (28) | 2018 | Hospital | 44 | 1423 | NS | Mixed | Adults | NS | NS | Retrospective | AKI 3.1% | **Moderate** |
| Martnez-García (77) | 2017 | ICU | 33 | 90 | NS | Septic shock | Pediatrics | Own | (SCr) ⃰ 1.5 or UO < 0.5 ml/g/h | Retrospective | AKI 36.7% | **Low** |
| Medina-Hernandez (27) | 2015 | Hospital | 6 | 13 | NS | Obstetric | Adults | NS | NS | Retrospective | AKI 46.2%,  severe AKI 23.1% | **Moderate** |
| Meza-Ayala (57) | 2018 | Hospital | 36 | 67 | 4 | ADHF | Adults | AKIN | SCr | Prospective | AKI 53.7%,  severe AKI 28.4% | **Moderate** |
| Moguel-González (76) | 2013 | ICU | 80 | 164 | 10 | Cardiac surgery | Adults | KDIGO | SCr or UO | Prospective | AKI 48.8% | **Low** |
| Molina-Valdes (26) | 2018 | ICU | 16 | 25 | NS | ARDS by A H1N1 | Adults | NS | NS | Prospective | AKI 62.5% | **Moderate** |
| Morales-Buenrostro (75) | 2014 | ICU | 17 | 56 | 8 | Multi-organ failure | Adults | AKIN | SCr or UO | Prospective | AKI 30.4% | **Low** |
| Muciño-Bermejo (74) | 2015 | ICU | 36 | 66 | NS | Cirrhosis | Adults | NS | NS | Retrospective | AKI 54.4% | **Moderate** |
| Olguin-Ramire  z (23) | 2016 | Hospital | 11 | 20 | 3 | Cerebrovascular | Adults | KDIGO | SCr | Retrospective | AKI 55.0% | **Moderate** |
| Olivas-Martine  z (22) | 2018 | Hospital | 24 | 49 | NS | Liver transplant | Adults | NS | NS | Retrospective | Severe AKI  49.0% | **Moderate** |
| Olivo-Gutierrez  (21) | 2016 | Hospital | 189 | NS | 50 | Mixed | Adults | NS | NS | Retrospective | Mortality  26.5% | **Moderate** |
| Olivo-Gutierrez  (21) | 2016 | Hospital | 198 | NS | 65 | Mixed | Adults | NS | NS | Retrospective | Mortality  32.8% | **Moderate** |
| Orozco-Méndez (58) | 2011 | ICU | 250 | 314 | NS | Obstetric | Adults | Own | UO < 0.5 ml/kg/h or < 500 ml/24 h | Retrospective | AKI 79.7% | **Moderate** |
| Ortiz-Rodriguez (25) | 2015 | Hospital | 6 | NS | 3 | Acute coronary  syndrome | Adults | NS | NS | Retrospective | Mortality  50.0% | **Moderate** |
| Ortiz-Rodriguez (24) | 2016 | Hospital | 11 | 78 | NS | Percutaneous coronary  intervention | Adults | Own | (SCr) ⃰ 1.25 or  ↑ ≥ 0.5 mg/dl | Retrospective | AKI 14.1% | **Moderate** |
| Palacios Moguel (59) | 2019 | ICU | 31 | 63 | 1 | Cardiac surgery | Adults | KDIGO | SCr or UO | Prospective | AKI 49.2%,  severe AKI 12.7% | **Low** |
| Perez-Cruz (16) | 2004 | Hospital | 13 | 182 | 1 | Obstetric | Adults | NS | NS | Retrospective | AKI 7.1% | **Moderate** |
| Perez-Jesus  (20) | 2017 | ICU | 42 | 64 | NS | Obstetric | Adults | NS | NS | Retrospective | AKI 65.6% | **Moderate** |
| Perez-Topete  (60) | 2016 | Hospital | 10 | 70 | 3 | Percutaneous coronary  intervention | Adults | Own | (SCr) ⃰ 1.25 or  ↑ ≥ 0.5 mg/dl | Retrospective | AKI 14.3% | **Moderate** |
| Quevedo-Gutierrez (18) | 2018 | Hospital | 129 | 388 | NS | Acute coronary  syndrome | Adults | KDIGO | SCr | Retrospective | AKI 33.2% | **Moderate** |
| Renteria-Diaz  (61) | 2017 | ICU | 25 | 66 | NS | Mixed | Adults | AKIN | SCr or UO | Retrospective | AKI 37.8% | **Low** |
| Reyes-Flandes (73) | 2017 | ICU | 31 | 91 | NS | Cardiac surgery | Pediatrics | pRIFLE | SCr or UO | Retrospective | AKI 34.1%,  severe AKI 18.7% | **Low** |
| Reyna-Blanco (17) | 2015 | ICU | 264 | 463 | 93 | Mixed | Adults | KDIGO | SCr | Retrospective | AKI 57.0%,  severe AKI 44.1% | **Moderate** |
| Salazar-Escalante (63) | 2005 | ICU | 9 | 40 | 3 | Obstetric | Adults | NS | NS | Prospective | AKI 22.5% | **Moderate** |
| Santibanez-Velazquez (72) | 2014 | ICU | 55 | 489 | NS | Mixed | Adults | RIFLE | SCr or UO | Prospective | AKI 11.2%,  severe AKI 5.9% | **Low** |
| Vazquez-Rodriguez (71) | 2016 | ICU | 63 | 1305 | 1 | Obstetric | Adults | AKIN | SCr or UO | Retrospective | AKI 4.8%,  severe AKI 1.4% | **Low** |
| Vicente-Hernandez (66) | 2017 | Hospital | 21 | 82 | NS | Surgery | Adults | KDIGO | SCr or UO | Retrospective | AKI 25.6%,  severe AKI 13.4% | **Low** |
| Villalobos-Arreola (29) | 2015 | Hospital | 47 | 353 | NS | Percutaneous coronary  intervention | Adults | KDIGO | SCr | Retrospective | AKI 13.3% | **Moderate** |
| Wong (68) | 2015 | ICU | 19 | 55 | 1 | Mixed | Adults | RIFLE | SCr or UO | Retrospective | AKI 34.5% | **Low** |
| Zaragoza (67) | 2011 | ICU | 48 | 163 | NS | Mixed | Adults | AKIN | SCr or UO | Prospective | Severe AKI 29% | **Low** |
| Cordova-Sanchez (87) | 2021 | ICU | 264 | 434 | 35 | Cancer | Adults | KDIGO | SCr or UO | Retrospective | AKI 56%, Mort AKI 13% | **Moderate** |
| Catalan (88) | 2022 | Hospital | 46 | 86 | 8 | Liver transplant | Adults | KDIGO | SCr or UO | Prospective | AKI 53%, Mort AKI 17% | **Low** |
| Chavez-Iñiguez (89) | 2023 | Hospital | 651 | NS | 123 | Mixed | Adults | KDIGO | SCr | Retrospective | Mort AKI 18.8% | **Low** |
| Chavez-Iñiguez (90) | 2023 | Hospital | 92 | NS | 45 | Mixed | Adults | KDIGO | SCr or UO | Prospective | Mort AKI 48.9% | **Low** |
| Chavez-Iñiguez (91) | 2021 | Hospital | 288 | NS | 45 | Mixed | Adults | KDIGO | SCr | Prospective | Mort AKI 15.6% | **Low** |
| Colin-Vazquez (92) | 2021 | Hospital | 22 | 100 | NS | Trauma | Adults | AKIN | SCr | Prospective | AKI 22% | **Moderate** |
| De la Vega-Mendez (93) | 2024 | Hospital | 49 | 516 | NS | Mixed | Adults | KDIGO | SCr | Retrospective | AKI 9.4% | **Low** |
| Martinez-Martinez (94) | 2020 | ICU | 15 | 75 | NS | Obstetric | Adults | AKIN | SCr | Retrospective | AKI 20% | **Moderate** |
| Medina-Gonzalez (95) | 2024 | ICU | 155 | NS | 87 | Mixed | Adults | KDIGO | SCr | Retrospective | Mort AKI 56.1% | **Low** |
| Ruiz-Gallardo (96) | 2023 | Hospital | 75 | NS | 7 | Mixed | Adults | KDIGO | SCr | Retrospective | Mort AKI 9.33% | **Low** |
| Vazquez-Rodriguez (97) | 2022 | ICU | 7 | 34 | NS | Obstetric | Adults | Own | SCr > 1.1 mg/dl | Retrospective | AKI 20.5% | **Moderate** |
| Vazquez-Rodriguez (98) | 2021 | ICU | 9 | 23 | NS | Obstetric | Adults | Own | NS | Retrospective | AKI 39.13% | **Moderate** |
| SCr: Serum Creatinine; AKI: Acute Kidney Injury; NS: Non-specified; ICU: Intensive Care Unit; KRT: Kidney Replacement Therapy; CRRT: Continuous replacement therapy | | | | | | | | | | | | |

Supplementary material G. AKI prevalence and AKI mortality by definition


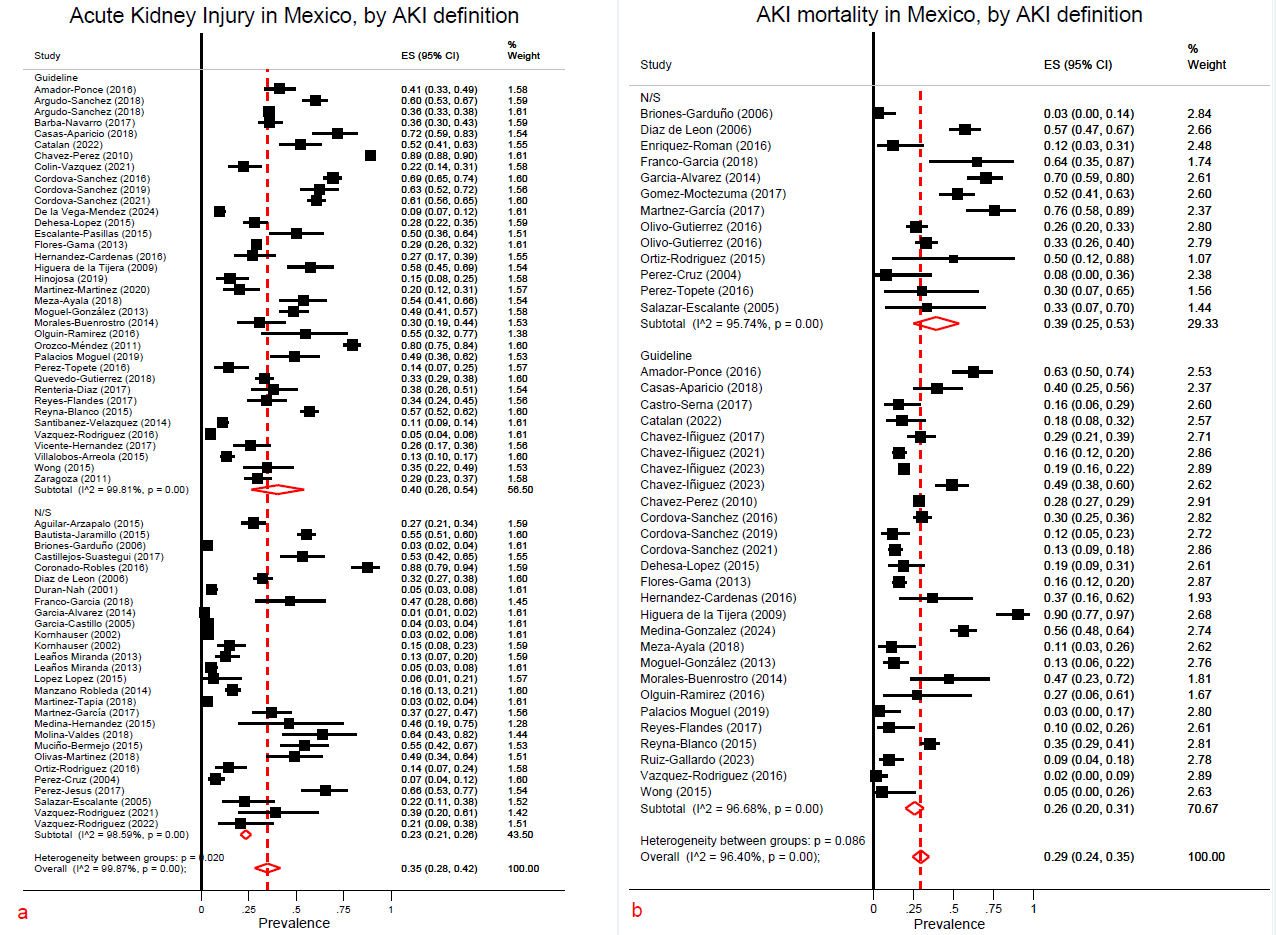


Supplementary material H. Summary of included studies in KRT information

| **Author** | **Year** | **Setting** | **Study population (n)** | |  | **Type of patient** | **Age of subjects** | **Outcome studied and assessment method** | | **Design** | **Main results Percentage (95% CI)** | **Overall risk of bias assessment** |
| --- | --- | --- | --- | --- | --- | --- | --- | --- | --- | --- | --- | --- |
|  |  |  | **KRT** | **AKI** | **KRT**  **mortality** |  |  | **KRT**  **Criteria** | **Modality** |  |  |  |
| Quevedo-Gutierrez (18) | 2018 | Hospital | 6 | 388 | 4 | Acute coronary syndrome | Adults | KDIGO by  SCr | KRT | Retrospective | KRT 1.5% | Moderate |
| Meza-Ayala (57) | 2018 | Hospital | 5 | 67 | NS | Heart Failure | Adults | NS | NS | Prospective | KRT 7.5% | Moderate |
| Cordova-Sanchez (82) | 2016 | ICU | 5 | 389 | 3 | Cancer | Adults | NS | IDH | Retrospective | KRT 1.3% | Low |
| Barba-Navarro (85) | 2017 | ICU | 7 | 233 | NS | Cardiac surgery | Adults | NS | NS | Prospective | KRT 3% | Low |
| Flores-Gama (70) | 2013 | ICU | 56 | 1096 | 30 | Cardiac surgery | Adults | Physician  criteria | KRT | Retrospective | KRT 5.1% | Low |
| Olguin-Ramirez (23) | 2016 | Hospital | 1 | 20 | NS | Cerebrovascular | Adults | NS | NS | Retrospective | KRT 5.0% | Moderate |
| Olivas-Martinez (22) | 2018 | Hospital | 8 | 49 | NS | Liver transplant | Adults | (SCr) ⃰ 2 or  KRT | NS | Retrospective | KRT 16.3% | Moderate |
| Hernandez-Lopez (30) | 2015 | ICU | 1 | 35 | NS | Mixed | Adults | NS | NS | Retrospective | KRT 4.0% | Moderate |
| Escalante-Pasillas (38) | 2015 | ICU | 8 | 52 | NS | Mixed | Adults | NS | NS | Prospective | KRT 15.4% | Moderate |
| Wong (68) | 2015 | ICU | 1 | 55 | NS | Mixed | Adults | NS | NS | Retrospective | KRT 1.8% | Low |
| Amador-Ponce (50) | 2016 | ICU | 11 | 155 | NS | Mixed | Adults | AKIN by UO  or SCr | NS | Prospective | KRT 7.1% | Moderate |
| Dehesa-Lopez (41) | 2015 | Hospital | 5 | 192 | NS | Mixed | Adults | NS | NS | Retrospective | KRT 2.6% | Moderate |
| Bautista-Jaramillo (47) | 2015 | ICU | 21 | 449 | NS | Mixed | Adults | NS | NS | Retrospective | KRT 4.7% | Moderate |
| Reyna-Blanco (17) | 201  5 | ICU | 33 | 463 | 18 | Mixed | Adults | NS | NS | Retrospective | KRT 7.1% | Moderate |
| Santibanez- Velazquez  (72) | 2014 | ICU | 7 | 489 | 5 | Mixed | Adults | Physician criteria | CRRT | Prospective | KRT 1.4% | Low |
| Avalos-Lopez (86) | 2017 | ICU | 219 | 2806 | 81 | Mixed | Adults | (SCr) ⃰ 3 or physician criteria | IHD 73.8%,  CRRT 26.2% | Retrospective | KRT 7.8%  (IHD 73.8%, CRRT 6.2%) | Low |
| Chavez-Perez (44) | 2010 | ICU | 361 | 5829 | 193 | Mixed | Adults | NS | IHD 49.7%,  CRRT 22.8%, PD 21.7%,  mixed 5.8% | Multicentric  prospective | KRT 6.2% | Low |
| Hernandez-Lopez (31) | 2014 | ICU | 2 | 54 | NS | Obstetric | Adults | NS | NS | Prospective | KRT 3.7% | Moderate |
| Perez-Cruz (16) | 2004 | Hospital | 3 | 182 | NS | Obstetric | Adults | NS | NS | Retrospective | KRT 1.6% | Moderate |
| Vazquez-Ro  driguez (71) | 201  6 | ICU | 1 | 1305 | NS | Obstetric | Adults | NS | NS | Retrospective | KRT 0.1% | Low |
| Briones-Garduño (84) | 2006 | ICU | 6 | 1272 | 1 | Obstetrics | Adults | NS | PD | Retrospective | KRT 0.5% | Moderate |
| Perez-Topete (60) | 201  6 | Hospital | 1 | 70 | NS | Percutaneos coronary  intervention | Adults | NS | NS | Retrospective | KRT 1.4% | Moderate |
| Diaz de Leon (52) | 2006 | ICU | 32 | 332 | 9 | Peritonitis | Adults | Absolute criteria, urea  > 150 or SCr > 5 mg/dl | PIRRT | Retrospective | KRT 9.6% | Moderate |
| Casas-Aparicio (83) | 2018 | ICU | 4 | 60 | NS | Pneumonia by influenza  A H1N1 | Adults | NS | NS | Retrospective | KRT 6.7% | Low |
| Hernandez- Cardenas (32) | 2016 | ICU | 7 | 70 | NS | Pneumonia by influenza A H1N1 | Adults | NS | NS | Prospective | KRT 10% | Low |
| Arevalo-Espinosa (55) | 2001 | ICU | 5 | NS | 4 | Cardiac surgery | NS | NS | NS | NS | Mortality  80% | Low |
| Gonzalez-Michaca (33) | 2000 | ICU | 24 | NS | 19 | Mixed | NS | NS | NS | NS | Mortality  79.2% | Low |
| Rivera-Solis (62) | 2018 | ICU | 31 | NS | 19 | Mixed | NS | NS | NS | NS | Mortality 61.3%,  recovery  35.5% | Low |
| Carrillo-Molina (46) | 2017 | ICU | 48 | NS | 33 | Mixed | NS | NS | NS | NS | Mortality  68.0% | Moderate |
| Pozos-Cortes (19) | 201  6 | ICU | 71 | NS | 37 | Mixed | NS | NS | NS | NS | Mortality  52.1% | Moderate |
| Olivo-Gutierrez (21) | 2016 | Hospital | 88 | NS | 25 | Mixed | NS | NS | NS | NS | Mortality 28.4%,  recovery  31.8% | Moderate |
| Olivo-Gutierrez (21) | 2016 | Hospital | 92 | NS | 40 | Mixed | NS | NS | NS | NS | Mortality 43.4%,  recovery  30.4% | Moderate |
| Canseco-Morales (54) | 2010 | ICU | 24 | NS | 17 | Mixed/Trauma/Burns | NS | NS | NS | NS | Mortality  70.8% | Low |
| Ibarra-Hernandez (69) | 2017 | Hospital | 10 | 27 | 0 | Pregnancy and CKD | Adults | NS | NS | NS | Mortality 0%,  recovery  20% | Low |
| Ibarra-Hernandez (69) | 2017 | Hospital | 18 | NS | 0 | Pregnancy and CKD | Adults | NS | NS | NS | Mortality 0%,  non-recover  y 44.4% | Moderate |
| Cordova-Sanchez (87) | 2021 | ICU | 4 | 264 | 35 | Cancer | Adults | NS | NS | Retrospective | KRT 1.5%  Mort KRT 25% | Low |
| Catalan (88) | 2022 | Hospital | 9 | 45 | 8 | Liver transplant | Adults | NS | NS | Prospective | KRT 20% | Low |
| Chavez-Iñiguez (88) | 2023 | Hospital | 211 | 651 | 123 | Mixed | Adults | NS | KRT (IHD, CRRT, PD) | Retrospective | KRT 32.4% | Low |
| Chavez-Iñiguez (89) | 2023 | Hospital | 17 | 92 | 45 | Mixed | Adults | NS | KRT (IHD, CRRT, PD) | Prospective | KRT 18.4% | Low |
| Chavez-Iñiguez (90) | 2021 | Hospital | 72 | 288 | 45 | Mixed | Adults | NS | KRT (IHD, CRRT, PD) | Prospective | KRT 25%  Mort KRT 27.7% | Low |
| Medina-Gonzalez (95) | 2024 | ICU | 100 | 155 | 87 | Mixed | Adults | NS | KRT (IHD, CRRT, PD) | Retrospective | KRT 64.5%  Mort KRT 39% | Low |
| Vazquez-Rodriguez (97) | 2022 | ICU | 2 | 7 | NS | Obstetric | Adults | NS | NS | Retrospective | KRT 28% | Moderate |
| Vazquez-Rodriguez (98) | 2021 | ICU | 1 | 9 | NS | Obstetric | Adults | NS | NS | Retrospective | KRT 11.1% | Moderate |
| SCr: Serum Creatinine; AKI: Acute Kidney Injury; NS: Non-specified; ICU: Intensive Care Unit; CKD: Chronic Kidney Disease | | | | | | | | | | | | |

Supplementary material I. a) KRT by hospital setting, b) KRT by year of publication


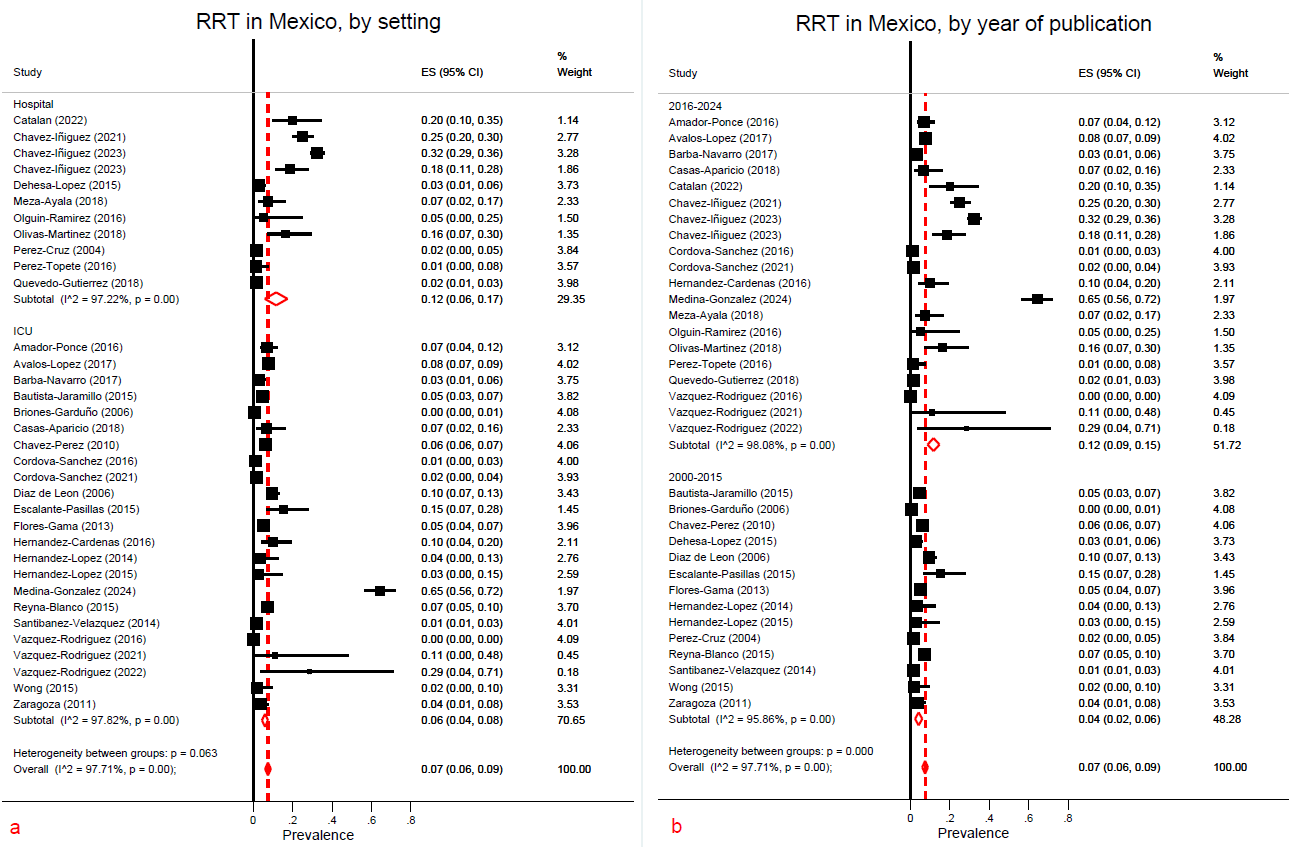


Supplementary material J. a) KRT mortality by hospital setting, b) KRT mortality by year of publication


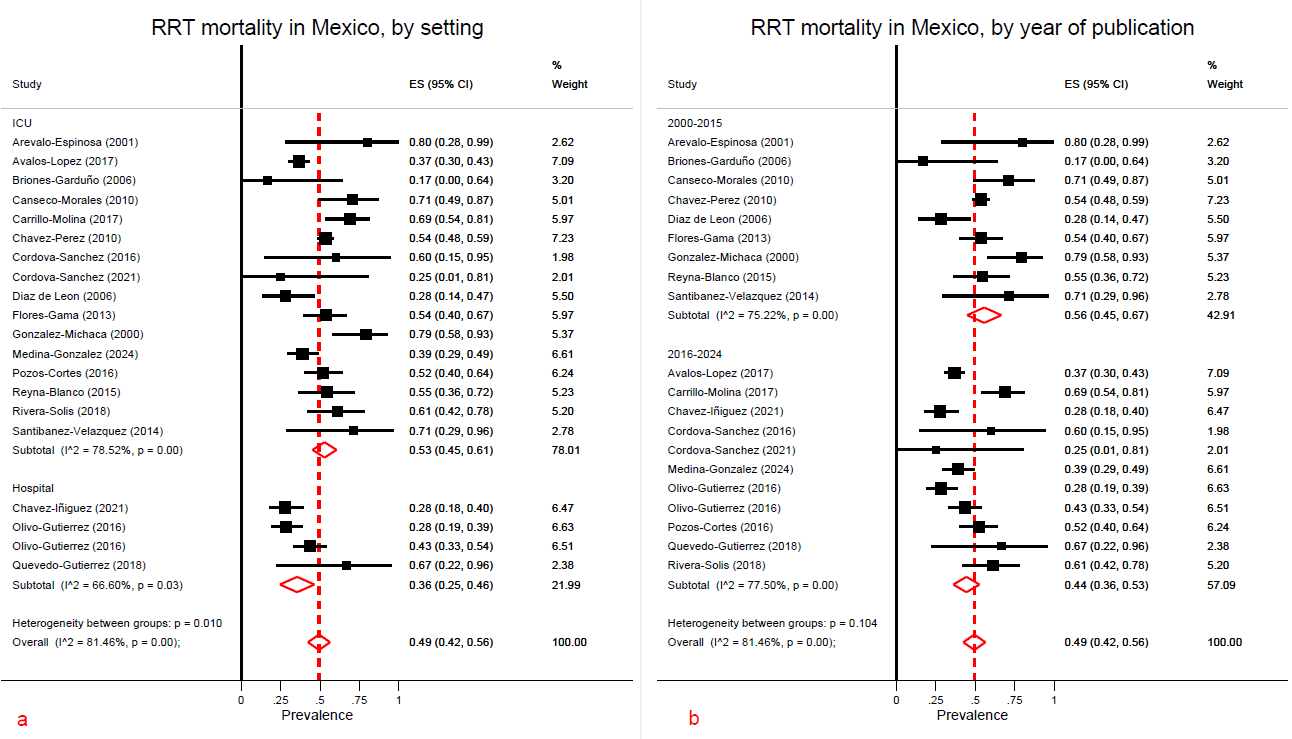

Supplement: Supplementary material 09_12_24.docx [file IRNF_A_2449573_SM4699.docx]
